# Effect of Carbon Nanotubes on Chloride Diffusion, Strength, and Microstructure of Ultra-High Performance Concrete

**DOI:** 10.3390/ma17122851

**Published:** 2024-06-11

**Authors:** Mahdi Rafieizonooz, Jang-Ho Jay Kim, Jin-Su Kim, Jae-Bin Jo

**Affiliations:** School of Civil and Environmental Engineering, Yonsei University, Yonsei-ro 50, Seodaemun-gu, Seoul 03722, Republic of Korea; mahdirafiei@yonsei.ac.kr (M.R.); kjinsu@yonsei.ac.kr (J.-S.K.); whwoqls@yonsei.ac.kr (J.-B.J.)

**Keywords:** carbon nanotubes (CNTs), ultra-high performance concrete (UHPC), mechanical properties, ponding test, life service prediction

## Abstract

This study delved into the integration of carbon nanotubes (CNTs) in Ultra-High Performance Concrete (UHPC), exploring aspects such as mechanical properties, microstructure analysis, accelerated chloride penetration, and life service prediction. A dispersed CNT solution (0.025 to 0.075 wt%) was employed, along with a superplasticizer, to ensure high flowability in the UHPC slurry. In addition, the combination of high-strength functional artificial lightweight aggregate (ALA) and micro hollow spheres (MHS) was utilized as a replacement for fine aggregate to not only reduce the weight of the concrete but also to increase its mechanical performance. Experimental findings unveiled that an increased concentration of CNT in CNT1 (0.025%) and CNT2 (0.05%) blends led to a marginal improvement in compressive strength compared to the control mix. Conversely, the CNT3 (0.075%) mixture exhibited a reduction in compressive strength with a rising CNT content as an admixture. SEM analysis depicted that the heightened concentration of CNTs as an admixture induced the formation of nanoscale bridges within the concrete matrix. Ponding test results indicated that, for all samples, the effective chloride transport coefficient remained below the standard limitation of 1.00 × 10^−12^ m^2^/s, signifying acceptable performance in the ponding test for all samples. The life service prediction outcomes affirmed that, across various environmental scenarios, CNT1 and CNT2 mixtures consistently demonstrated superior performance compared to all other mixtures.

## 1. Introduction 

Concrete is the foremost construction material globally, appreciated for its widespread availability, affordability, and versatility. Nevertheless, conventional concrete faces inherent constraints when aspiring to achieve structures that are simultaneously ultra-high-strength, lightweight, and durable. Consequently, the production of ultra-high-performance (UHP) concrete stands as a pivotal concern within the construction industry [1,2,3]. The specific compressive strength requirements for UHP concrete are contingent upon the structural demands of a project and the targeted performance characteristics [4,5]. However, UHP concrete, characterized by compressive strengths ranging from 80 MPa to 150 MPa or higher, has garnered significant attention in recent years [6,7,8]. This attention is driven by its remarkable load-bearing capacity, ability to diminish structural dimensions and weight, and heightened durability [9,10,11,12,13]. Attaining high-strength concrete involves employing diverse strategies, including optimizing mix designs, incorporating supplementary cementitious materials, maintaining a low water-to-cementitious material ratio (w/c), and utilizing chemical admixtures. Consequently, integrating certain admixture materials, such as Carbon nanotubes (CNTs), presents a distinctive opportunity to further enhance the performance of UHP concrete Top of Form [14,15]. 

CNTs have emerged as a promising material across diverse fields of science and engineering, owing to their exceptional mechanical, electrical, and thermal properties. As highlighted earlier, one notable domain where CNTs have demonstrated substantial potential is in the manufacturing of UHP concrete [16,17,18,19]. Integrating CNTs into concrete holds the promise of elevating its mechanical properties, enhancing durability, and facilitating the creation of inventive structural designs. The incorporation of CNTs in UHP concrete paves the way for the construction of resilient and sustainable infrastructures, capable of withstanding challenging conditions and ensuring long-term structural integrity. [20,21,22]. 

CNTs exhibited extraordinary mechanical properties, including high tensile strength, exceptional stiffness, and remarkable aspect ratios [23]. These characteristics position them as ideal candidates for reinforcing concrete at the nanoscale level. When uniformly dispersed within the concrete matrix, CNTs can bridge microcracks, contribute additional tensile strength, and enhance the overall mechanical performance of the material. Moreover, the excellent electrical conductivity of CNTs opens avenues for applications in sensing and monitoring structural health [24,25,26]. The conductive nature of CNTs allows for the development of intelligent and self-sensing concrete structures capable of detecting stress, strain, and temperature variations [27,28]. 

However, utilizing CNTs in UHP concrete effectively entails several significant challenges. Key issues include achieving uniform dispersion of CNTs within the concrete matrix to prevent agglomeration and ensuring efficient load transfer between the CNTs and the surrounding matrix [25,29]. Additionally, there are important considerations regarding the compatibility of CNTs with cementitious materials, long-term durability, and the economic feasibility of large-scale production [26]. Moreover, the incorporation of CNTs with various materials like silica fume, micro hollow spheres, and artificial lightweight aggregates in the production of UHP concrete could result in diverse characteristics of UHP concrete that warrant further investigation. Hence, further investigation is necessary to tackle this challenge and gain a better understanding of the performance of CNTs as additive materials in UHP concrete production.

In recent years, significant research efforts have been dedicated to comprehending the behavior and potential applications of CNTs in UHP concrete. These studies have delved into the mechanical properties, durability considerations, and structural performance of CNT-reinforced concrete. The objective is to surmount the challenges associated with CNT integration and lay the groundwork for the practical application of CNT-reinforced high-strength and UHP concrete in real-world construction projects. In addition to investigating mechanical properties, prior research has thoroughly explored the durability aspects of UHP concrete, with a specific focus on chloride penetration. Numerous studies have scrutinized the chloride ingress and penetration behavior of both conventional and UHP concrete [30,31]. These investigations have incorporated a variety of materials as replacements or admixtures, such as blast-furnace slag [32], polyvinyl-alcohol fiber [33], fly ash [34], and steel fibers [35]. Various approaches exist for examining chloride migration and chloride concentration in the concrete matrix [36]. A commonly employed method for long-term assessment is NT Build 443 [37], akin to ASTM C1543 [38] and ASTM C1556 [39]. Notably, the key distinction lies in the concentration of the ponding solution, where NT Build 443 employs a higher concentration (16.5 wt% NaCl) compared to the ASTM methods (15 wt% and 3 wt%, respectively). Hence, in the present study, NT Build 443 is employed to assess the chloride concentration profile following a 90-day ponding in a NaCl solution. 

Dehghan et al. [36] conducted a study to assess the suitability of X-ray microfluorescence for determining chloride diffusion coefficients in concrete chloride penetration experiments. The study employed two distinct test methods, namely NT Build 443 and NT Build 492 [40]. The results unveiled consistent trends across both test methods. In another study [41], ASTM C1556 is utilized to evaluate chloride binding and diffusion in slag-blended concrete mixtures. The findings of this investigation indicated that the incorporation of ground-granulated blast furnace slags enhanced chloride binding and reduced chloride diffusivity. Both NT Build 443 and NT Build 492 test methods were applied to ascertain the chloride diffusion coefficient in mortars incorporating blended cement [42]. The outcomes of this study demonstrated that the silver nitrate colorimetric technique provided adequate accuracy for determining the chloride penetration front. Additionally, it is established that the NT Build 492 and NT Build 443 test methods were suitable approaches for determining the chloride diffusion coefficient in mortars containing blended cement. Furthermore, a separate investigation [43] also employed both NT Build 443 and NT Build 492 methods to assess how microstructural properties impact the chloride diffusion resistance of alkali-activated materials. The findings indicated that both quantifying reaction products and establishing a correlation between chloride penetration and pore surface area suggested that physical chloride adsorption on the surfaces of C-A-S-H/N-A-S-H gels took precedence over chemical chloride binding. 

Recognizing that incorporating Carbon nanotubes (CNT) as an admixture in the production of ultra-high-strength (UHP) concrete alongside other replacement materials, such as silica fume (SF), silica powder (SPW), micro hollow sphere (MHS), and artificial lightweight aggregate (ALA), may exert varied influences on the mechanical properties and durability of the concrete, this study aims to assess the impacts of different CNT amounts as admixtures on the mechanical performance and chloride diffusion resistance of UHP concrete. The primary innovations of this paper include (1) an evaluation of the compatibility of CNTs with other cementitious materials and their long-term durability implications and (2) an economic feasibility analysis for the large-scale production of CNT-reinforced UHP concrete.

## 2. Materials and Mix Design

### 2.1. Materials

**Figure 1:**
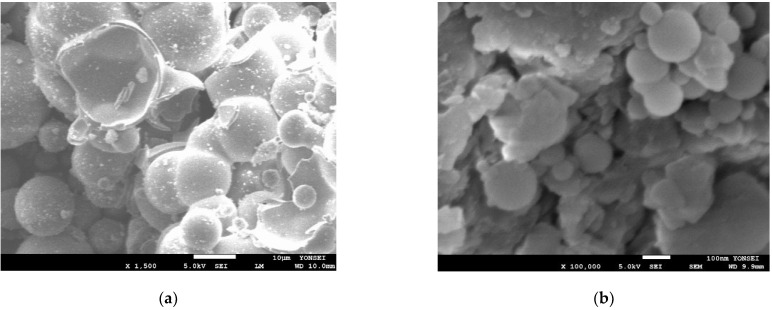
SEM Images of (**a**) MHS and (**b**) ALA.

Ultra-high-performance concrete is manufactured utilizing Ordinary Type I Portland cement sourced from Asia Cement Co. Ltd. in Seoul, Republic of Korea. The OPC’s characteristics include a specific gravity of 3.15, a soundness value of 10 mm, and a specific surface area of 2800 cm^2^/g. The chemical composition of OPC, micro silica powder (SPW), and micro hollow sphere (MHS) is outlined in Table 1. Upon examining the table, it is evident that MHS and SPW are predominantly composed of SiO_2_ (>81%), with minor quantities of other oxides. In contrast, CaO is the predominant oxide in OPC. For this study, SF (Grade 940U, Elkem, Norway) is employed as a 20% substitute for OPC, possessing a composition of 95% SiO_2_, a Loss On Ignition (LOI) at 950 °C of 1.9%, and a specific gravity of 2.2. Additionally, micro SPW (S-SIL 10, SAC, Ulsan, Republic of Korea), with a diameter ranging from 1 to 5 μm, served as a fine filler, the chemical composition of which is detailed in Table 1. Moreover, high-strength functional artificial lightweight aggregate (ALA), created by embedding TiO_2_ epoxy into the internal pores (~0.3–300 μm) of pristine ALA and coating the external surface simultaneously (detailed coating process information is available in [44]), was utilized as a partial replacement for fine aggregate. The ALA exhibited a density of 1.4 g/cm^3^ and a water absorption rate of 14.1%. Figure 1 displays the SEM images of ALA and MHS particles. The MHS employed in this investigation is a type of hollow glass micro-solid bubble, hereinafter referred to as the micro-hollow sphere, produced and marketed by 3M. This micro-solid bubble is a lightweight material utilized in various applications such as aircraft bodies, automobile bodies, and construction, owing to its low density, high strength, and outstanding insulation properties. Being a factory-manufactured product ensures consistent quality and a reliable supply, making it well-suited for construction materials. The physical properties of MHS are displayed in Table 2. Also, in the current work for achieving high compressive strength, reducing the amount of water, and having appropriate workability, a superplasticizer (SP) was utilized.

### 2.2. Carbon Nanotube

Diverse nanomaterials are being employed to enhance concrete performance, with carbon nanotubes (CNT) being one such material. Studies have indicated that incorporating an optimal amount of CNT can create nucleation sites during early hydration, leading to the formation of a dense hydrate structure and increased strength. Achieving uniform dispersion of nanomaterials, including CNTs, within the cement matrix is crucial for ensuring concrete performance. A previous method involved pre-dispersing CNTs and nanomaterials uniformly in a solvent (such as water or surfactant) before application [24,45,46,47,48]. This approach, utilized in this research, involved the use of CNTs at ratios of 0%, 0.025%, 0.05%, and 0.075% relative to the weight of Ordinary Portland Cement (OPC). The present study employed a unified CNT solution. The procedure for creating this unified CNT solution, developed in a prior study, involves combining water, CNT powder, and dispersant, detail information is available in Siahkouhi et al. [48].

### 2.3. Mix Proportion

The procedure employed in this study involved several steps. Initially, dry materials including OPC, SF, SPW, MHS, and ALA were mixed for 5 min. Subsequently, a solution comprising water, SP, and CNT solution is added and mixed for an additional 5 min. The resulting mixture is then shaped into samples: cubic molds measuring 50 mm × 50 mm × 50 mm for compressive strength tests and cylindrical molds sized 200 mm × 100 mm for indirect tensile strength tests. These samples were left to cure at a temperature of 25 ± 3 °C for 24 h.

Curing in this study is conducted using two methods. Following the initial 24-h curing period, the samples underwent either (1) high-temperature curing at 90 °C and 95% humidity for 2 days, followed by subsequent curing in normal room air conditions at 25 ± 3 °C, or (2) high-temperature curing at 90 °C and 95% humidity for 2 days, succeeded by curing in a 25 °C sodium chloride (NaCl) solution, mimicking the conditions of a ponding test. The latter method aimed to replicate ponding test conditions and assess its impact on the mechanical properties and microstructure of the concrete mixtures.

The samples were subjected to compressive and tensile strength tests on days 3, 7, and 28. After conducting the compressive strength tests on days 3 and 28, further analyses, including X-ray Diffraction (XRD) and Scanning Electron Microscopy (SEM) image evaluations, were performed to investigate the effects of incorporating Carbon Nanotubes (CNTs) into the concrete mixtures. Table 3 displays the current study’s concrete mix design. According to this table, the control mix serves as the baseline reference, containing standard proportions of water, OPC, SF, SPW, MHS, ALA, and SP in concrete. No CNTs are added to this mix, making it the control group. Similar to the control mix, the CNT 1 batch maintains the same proportions of water, OPC, SF, SPW, MHS, ALA, and SP. However, a small amount of Carbon Nanotubes (0.21 kg/m^3^) is introduced into the mix. Like the previous mixes, CNT 2 maintains consistent quantities of water, OPC, SF, SPW, MHS, ALA, and SP. In this mix, a moderate amount of Carbon Nanotubes (0.43 kg/m^3^) is included. The CNT 3 mix, akin to the others, has the same amounts of water, OPC, SF, SPW, MHS, ALA, and SP as the control mix. Notably, a higher concentration of Carbon Nanotubes (0.64 kg/m^3^) is added in this batch. Varied quantities of CNT in the CNT 1, 2, and 3 mixtures enable the investigation of diverse CNT concentrations’ impact on the concrete’s properties, potentially influencing its strength and durability.

## 3. Experimental Methods

### 3.1. Flow Test 

The flow table test is a method used to measure the workability and consistency of concrete. It is performed to measure the ability of the concrete mix to flow and self-compact under its weight without the need for mechanical vibration. The flow test is essential to ensure that the concrete can fill the formwork completely and uniformly without segregation or excessive bleeding. The flow test provides a quantitative measurement of the flow properties of concrete. Unlike the slump test, which provides a qualitative measure of workability, the flow table test gives a numerical value that can be compared and monitored over time. The measured diameter of the concrete spread on the flow table provides a numerical value representing the flow of the concrete mix. Hence, the flow test is conducted to analyze the workability of CNT mixtures.

### 3.2. Compressive and Tensile Strength

The compressive strength test of concrete is one of the most common and important tests performed to ensure the quality of concrete. It measures the ability of a concrete specimen to withstand axial loads or forces applied along the axis of the specimen without any significant deformation. The compressive strength of concrete is a crucial factor in determining its structural capacity and durability. In this study, to analyze the compressive strength of CNT concrete three cube concrete specimens (50 × 50 × 50 mm) were cast and cured for each batch at three different ages of curing including 3, 7, and 28 days. Two distinct curing regimes were employed for the concrete specimens. Following demolding, all specimens were placed in an oven at a high temperature (90 °C) with 95% humidity for 48 h for both curing regimes. In the first curing method, the samples were subsequently cured at room temperature (23 °C) in the air until the testing day. Conversely, the second method involved curing the samples in a salt solution comprising 165 g of NaCl in 1 L of distilled water until the testing day. This salt solution is chosen to replicate conditions akin to the salt ponding test, allowing for an analysis of compressive strength in a challenging environmental scenario.

The indirect split tensile strength of concrete is a vital property that influences the durability, crack resistance, and overall performance of concrete structures. Therefore for analyzing the tensile strength, in this study, three cylindrical specimens (200 × 100 mm) were cast and cured for each concrete mixture at three different ages of curing including 3, 7, and 28 days. The curing method for the tensile test mirrored the initial curing process used for the compressive strength test. After demolding, the specimens were placed in an oven for 48 h and subsequently stored at room temperature in the open air until the testing day.

### 3.3. Microstructure and Phase Composition Analysis

Scanning Electron Microscopy (SEM), coupled with energy-dispersive X-ray spectroscopy (EDS), provides powerful tools to identify different phases and chemical elements present in the concrete. This information is crucial for understanding the composition of concrete materials and their interactions, which can affect the concrete’s properties and durability. Hence, in this work, SEM with EDS is utilized to analyze the microstructure of concrete mixtures at 3 and 28 days of both curing conditions. Concrete powders were collected after conducting the compressive strength test. In this research, the Field Emission Scanning Electron Microscope (FE-SEM) 7800F-Prime machine is used for SEM and EDS. 

Knowledge of the mineralogical composition provides insights into the concrete’s behavior under various conditions, such as shrinkage, cracking, and long-term strength development. X-ray Diffraction (XRD) is a powerful analytical technique used to determine the chemical composition and crystallographic structure of materials, including concrete. In the context of concrete analysis, XRD is primarily employed to identify the mineralogical composition of the crystalline phases present in the concrete. In this study, the Rigaku XRD (Ultima IV) machine is used to analyze the chemical composition and crystallographic structure of CNT concrete. 

### 3.4. Accelerated Chloride Penetration 

The salt ponding test, following NT Build 443, is a widely recognized procedure for evaluating the chloride permeability of concrete [2,8,30,36,42,43,49]. This test assesses the ability of concrete to resist the penetration of chloride ions, which is crucial for the durability and longevity of concrete structures, especially in harsh environmental conditions [32]. The primary purpose of the salt ponding test is to determine the chloride permeability characteristics of concrete surfaces. It helps in identifying areas prone to chloride ingress, which can lead to various issues such as corrosion of reinforcement, deterioration of concrete, and compromised structural integrity [50]. Therefore, in this study, a salt ponding test based on NT Build 443 [37] is selected and conducted to analyze the performance of UHP concrete containing CNT against chloride penetration. 

Following the standard specifications, three cylindrical samples (100 × 200 mm) were meticulously cast for each concrete mixture. Following demolding, these samples underwent a high-temperature curing process in an oven set at 90 °C, coupled with high humidity (95%) for a duration of 2 days. Subsequently, the cylindrical specimens were meticulously divided into halves by making a perpendicular cut along the axis of each cylinder. One of these halves is designated as the testing specimen, exposing its cut surface to a solution of sodium chloride (NaCl). After this initial exposure, the test samples were submerged in a saturated solution of calcium hydroxide (Ca(OH)_2_) at a controlled temperature of approximately 23 °C. To prevent carbonation, the samples were placed in tightly sealed plastic containers, ensuring that the containers were filled to the brim with the solution. The following day, the mass in a surface-dry condition (m_sd_) is meticulously determined by weighing the test samples, marking a crucial step in the evaluation process. A section, at least 20 mm thick, is sliced from the remaining half of the cast cylinder, extending the test samples. This section is utilized to assess the initial chloride concentration (C_i_).

The immersion of the test samples in the saturated calcium Ca(OH)_2_ solution persisted until the mass in a surface-dry condition (m_sd_) exhibited minimal changes, not exceeding 0.1 mass % per 24 h. Subsequently, all faces of the test samples, except the one designated for exposure, were air-dried at room temperature until they reached a stable white-dry condition. A uniform epoxy coating, approximately 1 mm thick, is then meticulously applied to these surfaces. Following a curing period of 48 h, in adherence to the epoxy’s specified requirements, the coated test samples were re-immersed in the Ca(OH)_2_ solution until the m_sd_ stabilized, following the previously described criteria.

**Figure 2:**
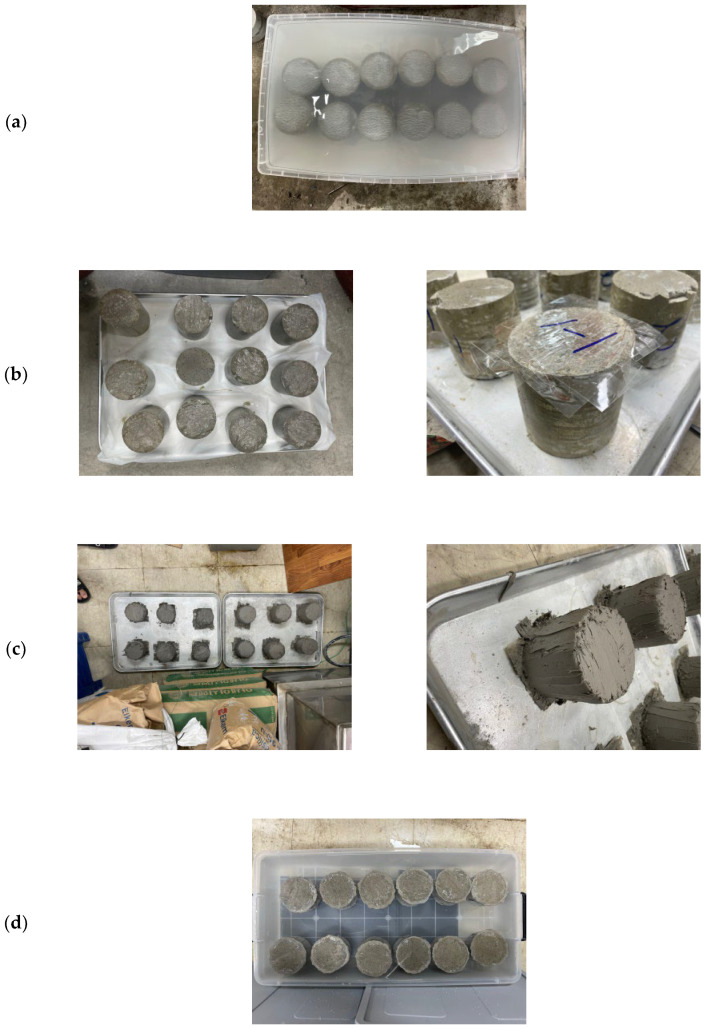
Ponding Test Procedure (NT-BUILD 443); (**a**) Samples in Ca (OH)_2_ solution, (**b**) Before Epoxy, (**c**) Applying the Epoxy, (**d**) Samples in NaCl solution.

Following the standard specifications, an aqueous sodium chloride (NaCl) solution is meticulously prepared, maintaining a concentration of 165 g ± 1 g NaCl per dm^3^ solution. This solution is utilized for a duration of 35 days (equivalent to 5 weeks) before being replaced by a fresh and pure NaCl solution. The concentration of NaCl in the solution is verified both before and after usage. The water bath, where the samples were placed, is carefully controlled, maintaining a temperature range between 21–25 °C, with an average target temperature of 23 °C (Figure 2). Additionally, the solution’s temperature is monitored once daily to ensure consistency throughout the testing period. Subsequently, the Ca(OH)₂ solution in the container used for water saturation was exchanged with the designated exposure liquid (NaCl solution), and the test samples were submerged in a surface-dry condition within the saline solution. It’s crucial to emphasize that the container is filled with the exposure liquid and tightly sealed. The ratio between the exposed surface area in square centimeters and the volume of the exposure liquid in cubic decimeters is maintained within a range of 20 to 80. 

The sealed container is then placed inside a temperature-controlled cupboard throughout the exposure period. This exposure phase lasted for a minimum of 90 days, during which the container is shaken once every week and the solution is replaced by a fresh, pure NaCl solution per 5 weeks (35 days). Detailed records, including the date and time of exposure commencement and conclusion, were diligently maintained. While the ponding test is capable of modeling chloride diffusion into concrete, it is still a long-term test. For low-quality concretes, the minimum exposure period is 35 days. For higher quality concretes, however, this period must be extended to 90 days or longer, just as for the salt ponding test [51].

Following a 90-day immersion process, the chloride profile is obtained by milling the material parallel to the exposed surface, necessitating a minimum of eight layers to be milled. The thickness of these layers should be adjusted based on the anticipated chloride profile, ensuring that at least six locations encompass the profile between the exposed surface and the depth reached by the chlorides. The chloride content soluble in acid within the specimens is evaluated following the NT BUILD 208 [52] standard. To determine the test results, including the superficial chloride concentration (C_s_) and the non-steady-state chlorides diffusion coefficient (D_ns_), Equation (1) is adjusted for the measured chloride content using linear regression analysis based on the least squares method.

(1) $$C_{\left(x,t\right)}=C_{s}-\left(C_{s}-C_{i}\right)*\mathrm{erf}\left(\frac{x}{\sqrt{4.D_{ns}.t}}\right)$$

In the given context: C_(x, t)_ represents chloride concentrations (in mass %) measured at a depth x” during the exposure time t”; C_s_ stands for the boundary condition at the exposed surface (in mass %); C_i_ denotes the initial measured chlorides concentration (in mass %); x” signifies the depth beneath the exposed surface (in meters); D_ns_ represents the non-steady-state chlorides diffusion coefficient (in m^2^/s); t” is the exposure time (in seconds), and erf refers to the Gauss error function.

### 3.5. Prediction of Service Life Model 

The chloride profiles obtained from concrete specimens subjected to an intense environmental condition (as in the ponding test) over 90 days offer a valuable chance to authenticate prevailing models predicting the service life. Several software packages currently exist for modeling service life in concrete structures, with Life-365 being one such model that forecasts chloride ingress and service life for reinforced concrete under chloride exposure [53]. Like certain other service-life prediction models, Life-365 operates under the assumption that concrete is entirely saturated [54]. However, in practice, reinforced concrete often undergoes wetting and drying cycles, and chloride penetration may not exclusively occur through diffusion. In this investigation, the Life-365 software is employed to formulate durability against salt damage, utilizing the results derived from the ponding test (NT Build 443). The design is customized for reinforced concrete (column dimensions: 600 mm × 600 mm), featuring cover thicknesses of 80 mm, 70 mm, and 60 mm. The assumed location is adjacent to the coastline. Furthermore, the external temperature to which the structure is subjected is presumed to align with the average monthly temperature in Korea for the year 2022.

As the migration of chloride ions in concrete follows a diffusion process, the prediction of chloride ion diffusion employs the diffusion equation derived from KDS 14 20 40 [55] and durability exceeding 30 years, as depicted in Equation (2). Equation (3) represents the most comprehensive form of the equation, incorporating adjustments for environmental conditions and chloride ion concentration on the surface.

t ≥ t_c_ = over 30 years

(2) $$D_{p}=\frac{D_{R}}{1-m}\;\left[\left(1-m\right)+m\left(\frac{t_{c}}{t}\right)\right]{\left(\frac{t_{R}}{t_{c}}\right)}^{m}$$

where: D_R_ denotes the chloride ion diffusion coefficient at the reference time (t_R_), t_R_ typically represents the reference time (commonly 28 days or 0.077 years), t_c_ signifies the diffusion coefficient reduction limit (typically set at 30 years), and m stands for the age coefficient relative to the age constant, representing its influence.

(3) $$C_{d}-C_{i}=(C_{s}-C_{i})\left(1-\mathrm{erf}\left(\frac{x}{2\sqrt{D.t}}\right)\right)$$

where: C_d_: chloride ion concentration (mass %) at the depth of x (m), C_i_: the initial chloride concentration (mass %), C_s_: the boundary condition at the exposed surface (mass %), D: diffusion coefficient of chloride ion (m^2^/s), t: the exposure time (s), and erf: error function

In this investigation, considering durability life spans of 200 years, the diffusion coefficient results obtained from the ponding test are incorporated into Equation (3), utilizing the standard chloride ion amounts specified for each exposure environment in KDS 14 20 40. This process is undertaken to determine the coating thickness for each environment. Durability-related considerations adhere to the concrete specifications outlined by the Korean Society of Civil Engineers (KSCE), as detailed in Table 4 and Table 5 [55]. It’s essential to highlight that, from both conditions (in the sea and near the sea), the maximum chloride concentration (as emphasized in Table 4 and Table 5) was chosen for each coastline to simulate the worst-case scenario. The recommended surface chloride amount and the limit chloride concentration of 1.2 (kg/m^3^) are taken into account. Additionally, by applying a safety factor, as per Equation (4) in KDS, considering the critical chloride concentration of 0.93 (kg/m^3^), the ensuing section presents the coating thickness for each environment.

(4) $$\gamma_{P}\times C_{d}\leq \emptyset_{K}\times C_{lim}$$

where: γ_P_ is an environmental factor for salt damage, generally 1.11, ϕ_K_ is the durability reduction factor for salt damage, generally 0.86, C_lim_ is critical chlorine ion concentration at the start of reinforcing bar corrosion (1.2 kg/m^3^), and C_d_ is the predicted value of chlorine ion concentration at the reinforcing bar location.

## 4. Results and Discussion

### 4.1. Fresh Properties

**Figure 3:**
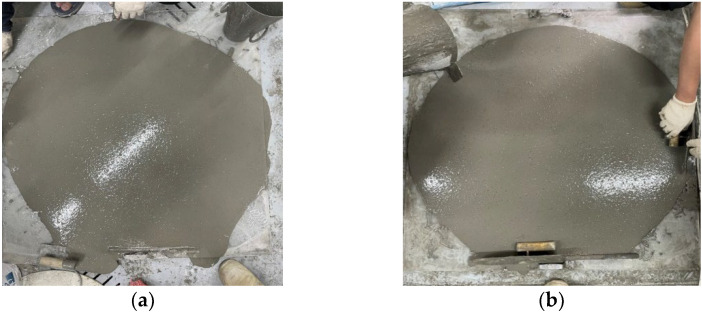
Flow test results of (**a**) Control, and (**b**) CNT2.

The outcomes of the flow test, illustrated in Figure 3, indicated that the incorporation of CNT as an admixture in the concrete matrix had a marginally positive impact on the fresh performance and flowability of the CNT concrete mixtures. All samples exhibited a flow test result higher than 100 cm; nevertheless, mixtures containing CNT as an admixture displayed slightly improved flowability compared to the control mixture. This enhancement may be attributed to the effective dispersion of CNTs, which can enhance concrete flowability by acting as a lubricating agent [56]. This reduces internal friction among particles, facilitating smoother flow in the concrete mix. However, the influence of adding CNTs on the workability of the CNT concrete mixtures is negligible. It’s important to note that substituting MHS for natural fine aggregate, given its spherical shape and micro particle size, led to an improvement in the flowability of all mixtures. Conversely, incorporating ALA as a partial replacement for fine aggregate, owing to its sharp edges and irregular shapes, caused a decrease in the flowability of concrete mixtures. However, in this study, through the use of sufficient superplasticizers, a well-designed mix proportion, and careful mixing procedures, suitable flowability is attained for all mixes.

### 4.2. Compressive Strength

**Figure 4:**
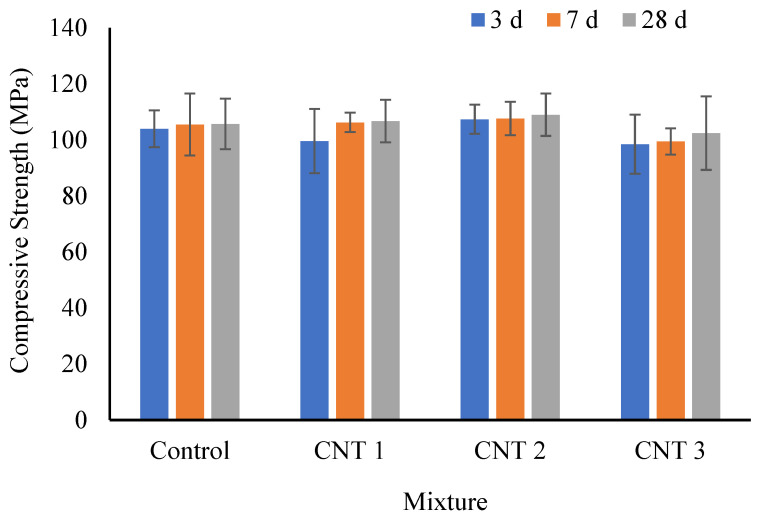
Compressive strength test results in normal curing conditions.

The compressive strength results under standard curing conditions for three different curing times are depicted in Figure 4. At the 3-day mark of standard curing, the compressive strength values for the control, CNT1, CNT2, and CNT3 mixtures were 103 MPa, 100 MPa, 107 MPa, and 100 MPa, respectively. Notably, there were no significant changes in compressive strength after the initial 3-day period of standard curing. Upon reaching the 7-day point with the standard curing method, the compressive strength values for the same mixes were 105 MPa, 106 MPa, 108 MPa, and 100 MPa, respectively. Comparing these results to the 3-day data, it becomes evident that all samples experienced a slight increase in compressive strength. This enhancement can be attributed to the ongoing hydration process and the formation of calcium silicate hydrate gel (C-S-H gel) within the concrete matrix, contributing to strength development over time [54,57]. The data from Figure 4 indicates that the compressive strength values for the same mixtures at the 28-day mark of the standard curing period were 106 MPa for the control mix, 107 MPa for CNT1, 109 MPa for CNT2, and 102 MPa for CNT3 mixture, respectively. Upon analysis of these results, it is observed that there was a modest increase in the compressive strength of all mixtures after a 28-day curing period compared to the results at 7 days. This improvement also can be attributed to the ongoing process of hydration extending up to the 28-day curing time.

Nevertheless, an examination of the data reveals that increasing the CNT content in CNT1 and CNT2 mixtures led to a slight improvement in compressive strength compared to the control mix. This enhancement may be attributed to the formation of bridges by CNTs between particles and cement paste within the concrete matrix. Conversely, the CNT3 mixture exhibited a decrease in compressive strength with the rise in CNT content as an admixture. It needs to be considered that the tendency of CNTs to form agglomerates, driven by strong van der Waals forces, becomes apparent [58,59,60]. When CNTs are inadequately dispersed, these agglomerates can create localized weak points in the concrete, diminishing its overall strength. This phenomenon likely accounts for the reduction in compressive strength observed in the CNT3 mixture compared to the other mixes. It suggests that the dispersion process of CNT in the CNT3 mixture was incomplete, resulting in the formation of localized weak points in the concrete matrix and a subsequent decrease in compressive strength.

**Figure 5:**
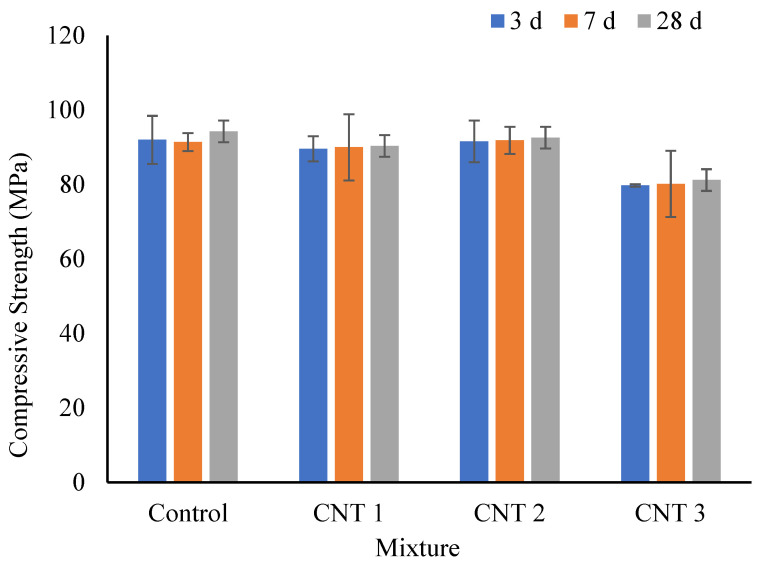
Compressive strength test results in salty curing condition.

Figure 5 illustrates the compressive strength results under salty curing conditions. At the 3-day mark of exposure to the salty curing environment, the compressive strength values for the control, CNT1, CNT2, and CNT3 mixes were 92 MPa, 90 MPa, 92 MPa, and 80 MPa, respectively. Notably, these findings indicate a decrease in compressive strength for the CNT3 mixes compared to the other formulations, aligning with observations from the normal curing condition. Additionally, after 7 days of exposure to salty curing conditions, there was no significant change in the compressive strength of all mixtures. Specifically, the compressive strength values for the same mixes were 91 MPa, 90 MPa, 92 MPa, and 80 MPa, respectively. Furthermore, upon reaching the 28-day mark in the salty curing conditions, the compressive strength values for the same mixes were 94 MPa, 90 MPa, 93 MPa, and 82 MPa, respectively.

From the results, it is evident that there is no substantial difference in compressive strength at various salty curing periods. However, it is crucial to emphasize that the compressive strength values at 3, 7, and 28 days in the salty curing environment were considerably lower than those observed under similar normal curing periods. This notable difference can be attributed to the presence of salts, particularly chloride ions [41]. These salts have the potential to interfere with the formation of C-S-H gel, a critical factor for the strength development of concrete. In the context of salty curing conditions, the impact on the formation of C-S-H gel is more pronounced, contributing to the observed reduction in compressive strength when compared to the results obtained under normal curing conditions. Moreover, salts have the capability to directly attack the cement paste, causing the leaching of calcium hydroxide and disrupting the paste’s structure. Consequently, this can result in a weaker matrix and lower compressive strength [43]. Additionally, salty curing conditions may elevate the porosity of the concrete. The presence of salts can induce the formation of additional pores or capillaries, thereby weakening the concrete structure and diminishing its overall strength [34,61]. Lastly, the presence of salts can disrupt the hydration reactions of cement, impeding the formation of desired crystalline phases. This interference can have a negative impact on the development of strength over time. 

### 4.3. Tensile Strength

**Figure 6:**
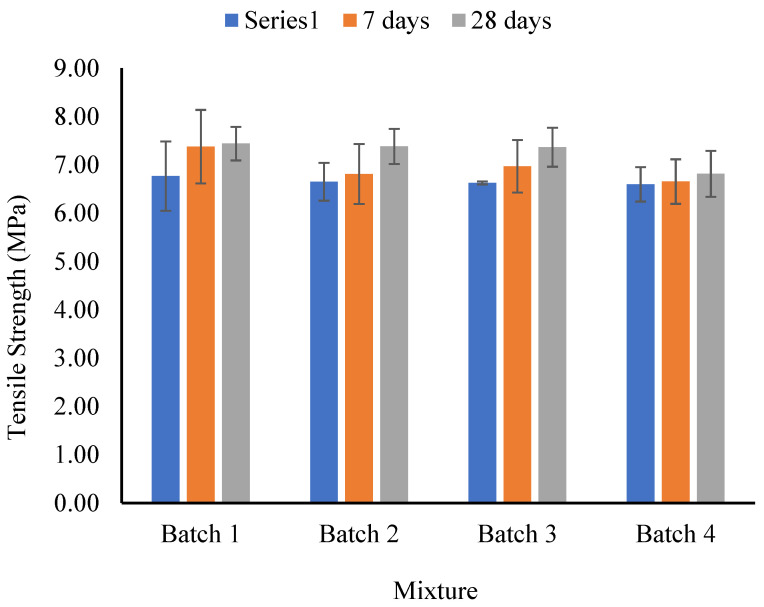
Indirect tensile strength test results in normal curing conditions.

Figure 6 presents the outcomes of the indirect spilite tensile strength test. At the 3-day mark of normal curing, the tensile strength values for the control, CNT1, CNT2, and CNT3 mixtures were 6.77 MPa, 6.65 MPa, 6.62 MPa, and 6.60 MPa, respectively. The results indicate a slight decrease in tensile strength with an increase in the amount of CNT as an admixture. This trend persists during the 7-day curing period, where the indirect tensile strength for the same mixtures was 7.38 MPa, 6.81 MPa, 6.97 MPa, and 6.65 MPa, respectively. The findings affirm the influence of elevated CNT content in diminishing the slight tensile strength of the concrete mixtures. At the 28-day mark of normal curing conditions, the indirect tensile strength for the same mixtures was 7.44 MPa, 7.38 MPa, 7.36 MPa, and 6.81 MPa, respectively. Similarly, it can be observed that the same trend persisted for tensile strength after 28 days of normal curing conditions. 

The impact of incorporating CNT as an admixture into high-performance concrete on compressive and tensile strength is subject to various factors. The observation of a slight increase in compressive strength alongside a slight decrease in tensile strength can be attributed to the following considerations [16,18,33]: Firstly, achieving a uniform dispersion and alignment of CNTs within the concrete matrix is paramount. Suboptimal dispersion or agglomeration of CNTs may lead to non-uniform properties. In terms of tensile strength, the presence of agglomerates or uneven distribution can create weak points, resulting in an overall decrease in tensile performance [14]. Secondly, while CNTs can augment compressive strength by serving as additional bridges and reinforcing the concrete structure, their interaction with the cementitious matrix may vary. The establishment of effective bonding between CNTs and the matrix is crucial for load transfer. If this bonding is not well-established, it may contribute to a reduction in tensile strength [15]. Lastly, the addition of CNTs can influence the porosity of the concrete. In cases where nanotubes are not well-dispersed, they may form clusters or induce the creation of additional pores, particularly at the interfaces between the nanotubes and the cement matrix. This, in turn, can adversely affect tensile strength.

Table 6 presents the results of the current study alongside findings from previous research. It is important to note that all properties of the UHP concrete in these studies are compared to those of the control mix concrete. As shown in this table, an increase in the water-to-binder ratio led to a slight reduction in compressive strength. An increase in the water-to-binder ratio typically results in a slight reduction in compressive strength due to dilution of the cement paste and increased porosity within the concrete matrix. This diminishes the binding properties of the cement paste and creates pathways for crack propagation, ultimately weakening the overall strength of the concrete. However, the type of nanomaterial and additive admixture significantly influenced the mechanical properties of UHP concrete.

### 4.4. SEM Investigation

**Figure 7:**
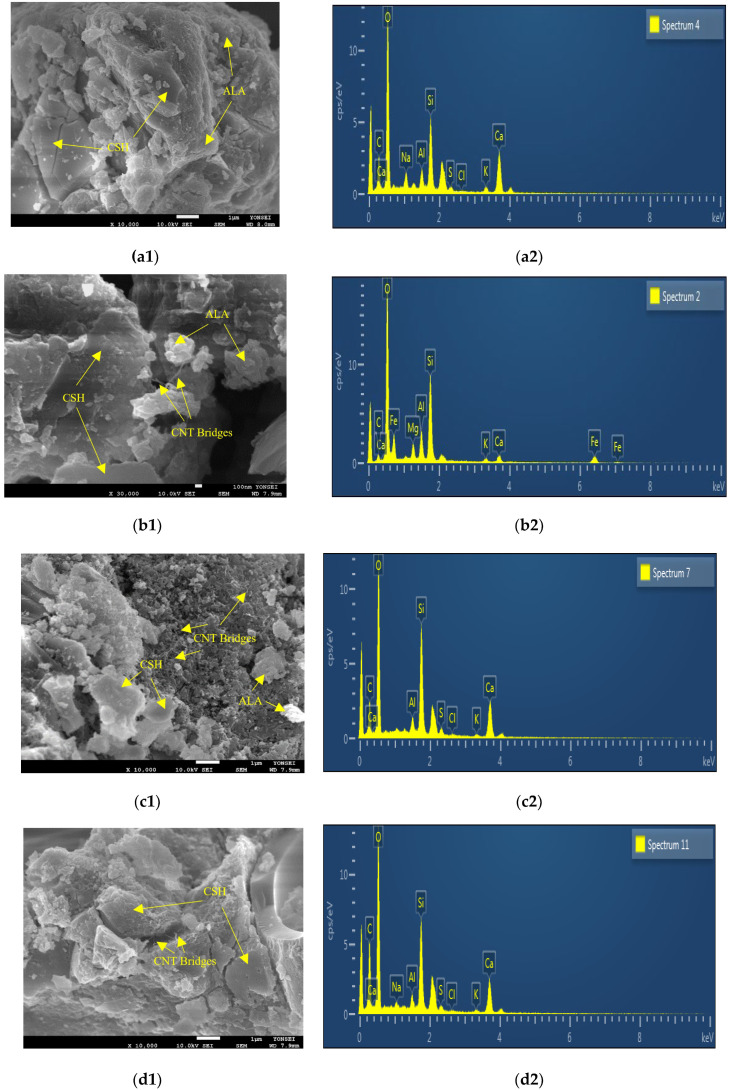
SEM images and EDS spectrum after 3 days of curing period for samples (**a**) control, (**b**) CNT1, (**c**) CNT2, and (**d**) CNT3.

The results obtained from Scanning Electron Microscopy (SEM) and energy dispersive X-ray spectroscopy (EDS) conducted during standard curing conditions over 3 days are illustrated in Figure 7. SEM analysis revealed that an increased content of CNTs as an admixture to the concrete resulted in the formation of bridges within the concrete matrix. These nanoscale bridges serve as reinforcement, creating a network within the cementitious matrix [47]. This network significantly improves the distribution and transfer of loads, thereby enhancing the overall strength performance of the concrete. Moreover, CNTs contribute to the reduction of microcracks and the enhancement of material toughness. Additionally, CNTs play a role in expediting the hydration process of cement particles, leading to the formation of a denser and more compact matrix. This accelerated curing process results in achieving higher compressive strength within a shorter timeframe. It can be asserted that, during the initial 3 days of curing, the presence of CNTs facilitates the formation of C-S-H gel in CNT1 and CNT2 mixtures. Conversely, in the case of the CNT3 mix, improper dispersion processes may lead to the creation of agglomerates. These agglomerates can form localized weak points in the concrete, diminishing its overall strength and durability [26]. Therefore, the effectiveness of CNTs in enhancing the concrete’s properties is contingent on a thorough dispersion process, emphasizing the importance of optimized mixing techniques for optimal results. 

Based on the outcomes derived from the energy dispersive X-ray spectroscopy (EDS) map sum spectrum, it is evident that O, Si, C, and Ca constitute the primary elements in the structure of all mixtures, albeit in varying proportions. These findings align seamlessly with the X-ray diffraction (XRD) results, wherein Quartz (SiO_2_) and Hatrurite (Alite: Tricalcium silicate: Ca_3_SiO_5_) emerge as the predominant crystalline phases. Furthermore, the results indicated a marginal increase in the amount of C with the escalation of CNT content as an admixture to the concrete matrix [15,62]. In summary, the SEM results, following a 3-day curing period, reveal a subtle structural divergence in CNT mixtures attributable to the presence of CNTs. However, in contrast, the EDS results suggest no significant disparity in the composition of all mixtures.

**Figure 8:**
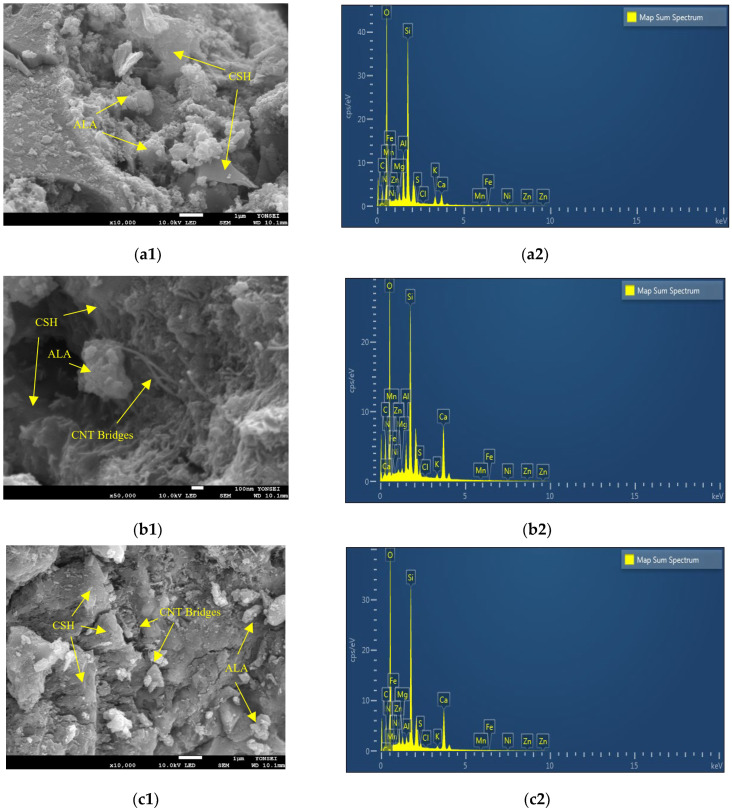

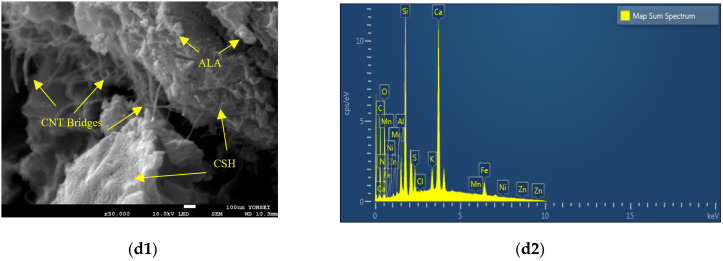
SEM images and EDS maps after 28 days of curing period for samples (**a**) control, (**b**) CNT1, (**c**) CNT2, and (**d**) CNT3.

Consistent findings were observed in both the scanning SEM images and EDS maps after a 28-day curing period (Figure 8). This suggests that, under standard curing conditions for 28 days, there was a modest increase in compressive strength attributed to the formation of bridges between the cement paste and particles (ALA and MHS). Notably, SEM images indicate a higher prevalence of bridges in samples subjected to a 28-day curing period compared to those cured for 3 days. 

The SEM images further indicated that with an increase in the quantity of CNT as an admixture, there was a noticeable augmentation in both the number and volume of bridges formed within the concrete matrix. Additionally, the inclusion of silica fume (SF) as a partial substitute for OPC enhances the potential for the secondary formation of C-S-H gel, particularly noticeable after extended curing periods (beyond 90 days). Consequently, the production of additional C-S-H gel becomes imperceptible beyond the 28-day mark.

The energy dispersive EDS map spectrum results for all mixtures after a 28-day curing period demonstrated a composition nearly identical to their 3-day counterparts. This uniformity is likely attributable to the absence of substantial changes in the structure and composition of all mixtures following the specified curing period. In each sample, O, Si, C, and Ca remained the primary elements, in line with the EDS map spectrum results. However, there is a slight increase in the percentage of C with the escalation of CNT content as an admixture [47,56]. These outcomes are consistent with the findings of XRD analysis, where quartz and hatrurite persist as the principal crystalline phases in the structure of all mixtures.

### 4.5. XRD Analysis

**Figure 9:**
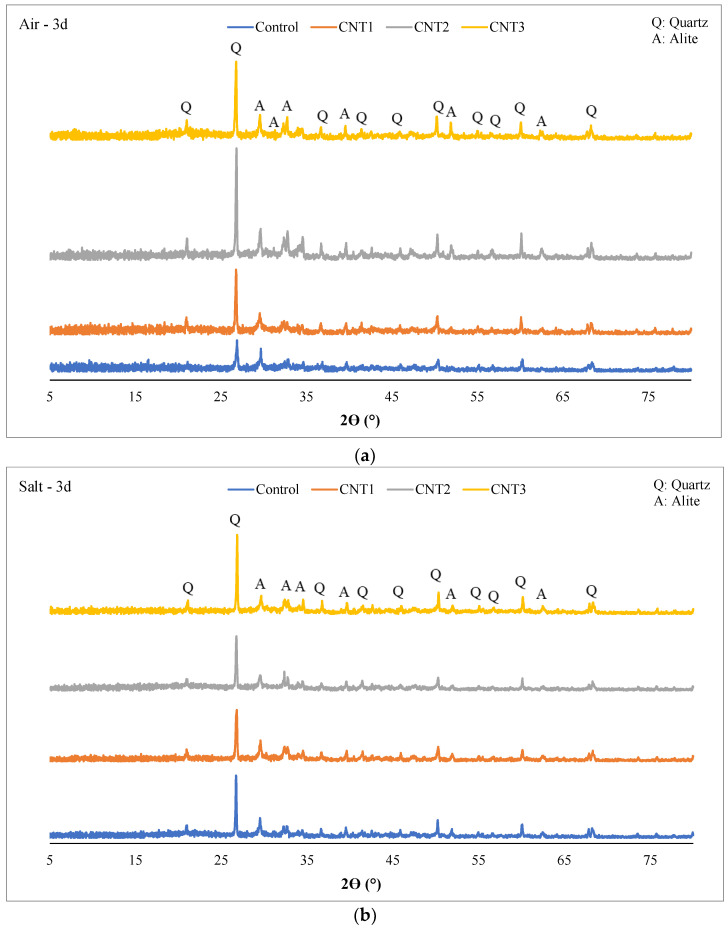
XRD Analysis of (**a**) 3d—Air curing, and (**b**) 3d—Salt curing condition.

**Figure 10:**
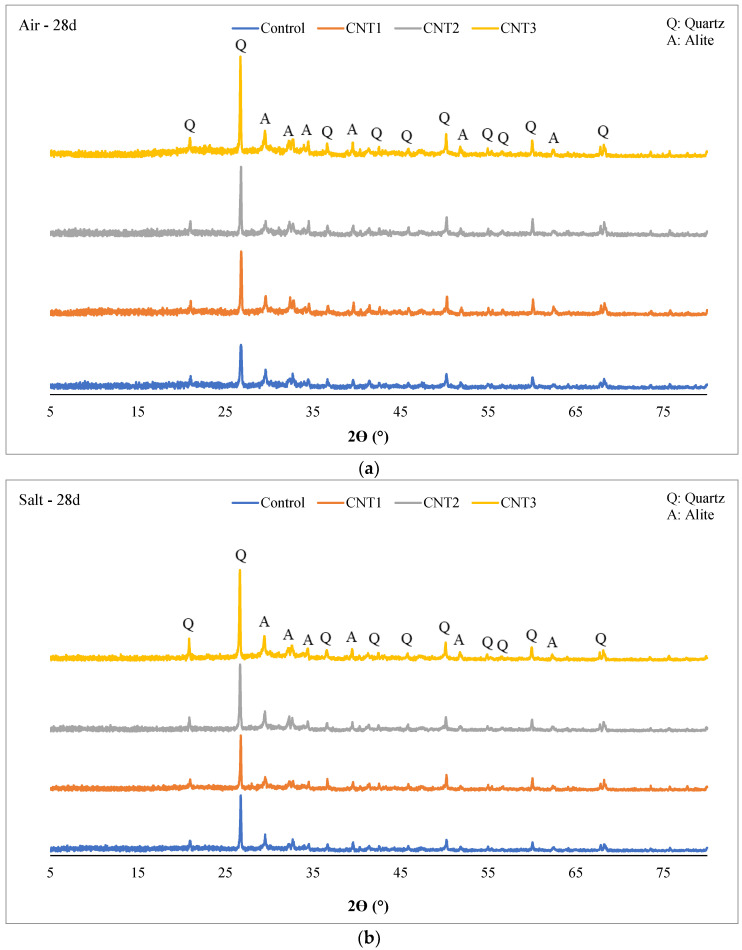
XRD Analysis of (**a**) 28d—Air curing, and (**b**) 28d—Salt curing condition.

The X-ray Diffraction (XRD) patterns depicted in Figure 9 and Figure 10 showcase concrete mixtures incorporating varying amounts of carbon nanotubes (CNT) at both 3 days and 28 days of the curing period. In all samples, after both 3 days and 28 days of curing, the primary crystalline phases identified include Quartz (SiO_2_) and Alite (Tricalcium silicate: Ca_3_SiO_5_).

Several factors may contribute to the presence of only these two phases:

1. Quartz is a common crystalline phase found in aggregates, particularly if natural aggregates or silica-based materials are utilized in the concrete mix.
2. Alite (Tricalcium silicate), a fundamental component of OPC, is expected in UHPC containing OPC.
3. Some materials in the mix may exist in an amorphous or non-crystalline form, and XRD is less sensitive to such phases.
4. The materials employed, including silica fume and silica powder, consist of extremely fine particles. Consequently, XRD may not identify them as distinct crystalline phases.
5. The mix composition may favor non-crystalline or amorphous forms, resulting in fewer distinguishable crystalline peaks.
6. XRD’s sensitivity limits might preclude the detection of phases present in small quantities or exhibiting weak diffraction signals.
7. A homogenized mix design could lead to the dominance of main crystalline phases, posing challenges in identifying minor phases.

It’s essential to recognize that the absence of certain phases in the XRD analysis doesn’t necessarily imply their complete nonexistence in the concrete mix. Some phases may exist in lower concentrations, or their diffraction peaks may be overshadowed by dominant phases. 

The inclusion of SF as a partial substitute for OPC in concrete has the potential to influence the XRD pattern, impacting the 2θ values of diffraction peaks. However, these alterations are typically subtle, particularly for primary crystalline phases like quartz. Notably, the prevalence of Alite, a significant contributor to early strength development in concrete, was higher in all mixes after 3 days of air curing compared to salt curing, as illustrated in Figure 9. In contrast, the abundance of quartz was greater in 3-day salt-cured samples than in their air-cured counterparts. This pattern persisted across all mixtures and likely serves as a key factor contributing to the augmented compressive strength observed in air-cured samples relative to their salt-cured counterparts.

The XRD analysis results at the 28-day mark under both curing conditions (as illustrated in Figure 10) displayed negligible differences compared to the 3-day curing period. Across all mixtures, the prevalence of quartz during the 28-day salt curing conditions surpassed that in the 28-day air curing conditions. In contrast, the concentrations of Alite in all mixtures were higher in the 28-day air curing conditions compared to the 28-day salt curing conditions. This disparity sheds light on the compressive strength results for these mixtures, where, after 28 days of air curing, the compressive strength notably exceeded that of the 28-day salt curing. The increased content of Alite in the air curing conditions likely contributed to the enhanced compressive strength through this curing method. Furthermore, it is essential to acknowledge that after 28 days of both curing methods, no new crystalline phases emerged in any of the mixtures. This suggests the absence of pozzolanic activity within the initial 28-day curing period. To induce pozzolanic activity, an extended curing period exceeding 90 days is required.

### 4.6. Ponding Test Results

**Figure 11:**
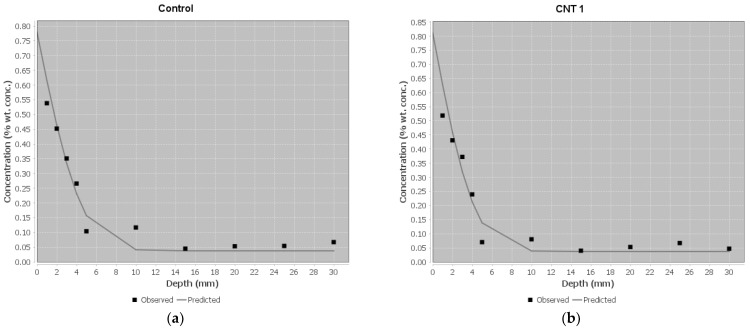

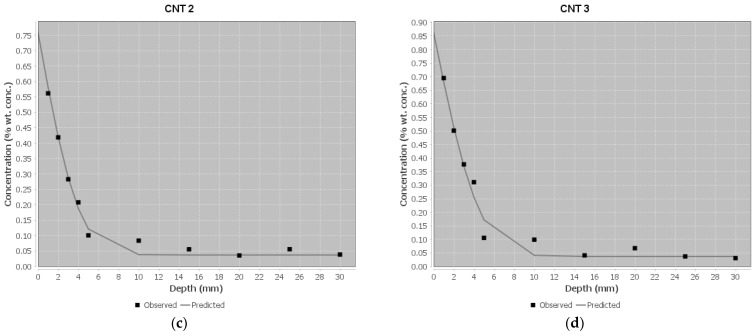
The regression Analysis based on the ponding test results for (**a**) Control, (**b**) CNT1, (**c**) CNT2, and (**d**) CNT3.

The findings from the ponding test are summarized in Table 7 and visually represented in Figure 11. In this context, C_s (mass%) denotes the boundary condition at the exposed surface, C_i (mass%) signifies the initial chloride concentration measured on the concrete slice, D_a (m^2^/s) represents the effective chloride transport coefficient, and t (days) denotes the exposure time. Analysis of the ponding test results reveals that, for all samples, the effective chloride transport coefficient (D_a) remains below the standard limitation of 1.00 × 10^−12^ m^2^/s. This suggests that the performance of all samples in the ponding test falls within an acceptable range. However, a closer examination of the results reveals that the chloride transport coefficient decreases with an increase in the amount of CNT up to 0.05% (as observed in CNT1 and CNT2 mixes compared to the control mix). Conversely, for the CNT3 mixture, there is an increase relative to the control mix. This trend aligns with the compressive and tensile strength outcomes, where the strength of the CNT1 and CNT2 mixtures surpassed that of the CNT3 mixture.

The primary factor influencing this trend is attributed to the incomplete dispersion process of CNT in the CNT3 mixture, resulting in the formation of localized weak points in the concrete matrix and an ensuing increase in permeability. Conversely, the well-dispersed CNT in the CNT1 and CNT2 mixes contributes to an elevated number of bridges between the cement paste and ALA and MHS particles, leading to decreased permeability and, ultimately, a reduced effective chloride transport coefficient in these two mixtures. 

Considering the C_s value, derived from the contact point of the predicted curve with the Y-axis in Figure 11, which represents the chloride mass on the surface of each mixture, the results correlate with those of the chloride transport coefficient. In summary, all mixtures demonstrate acceptable performance when compared to standard limitations. Notably, the CNT2 mixture stands out, exhibiting the most favorable performance among all mixes.

### 4.7. Life Service Prediction

**Figure 12:**
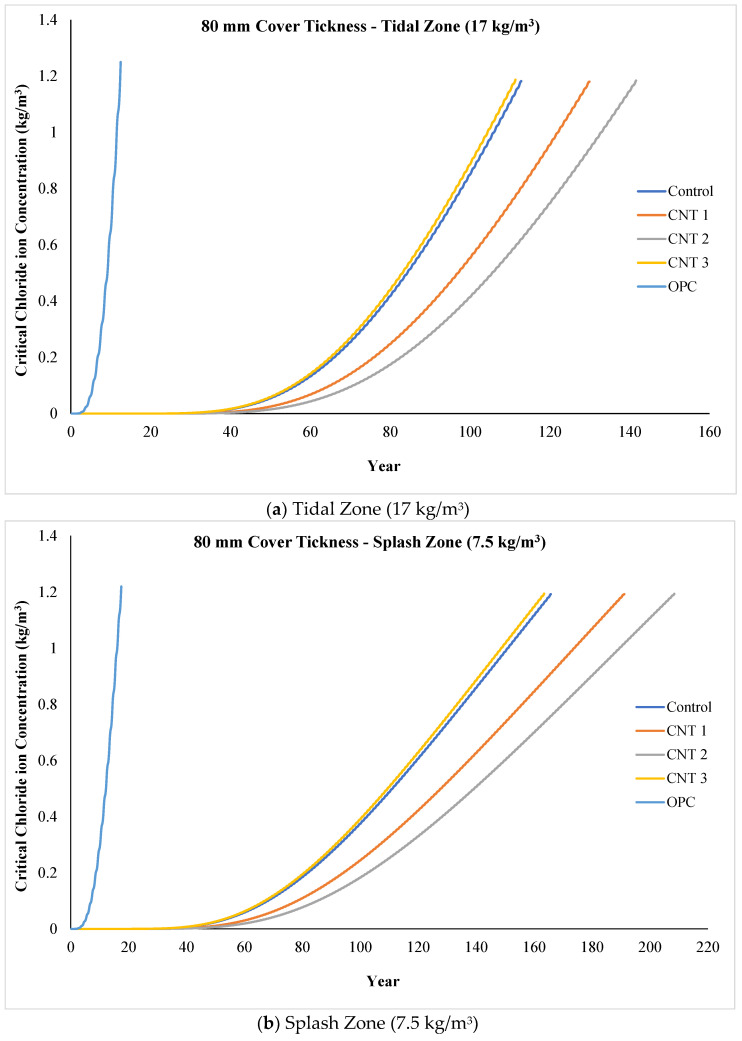

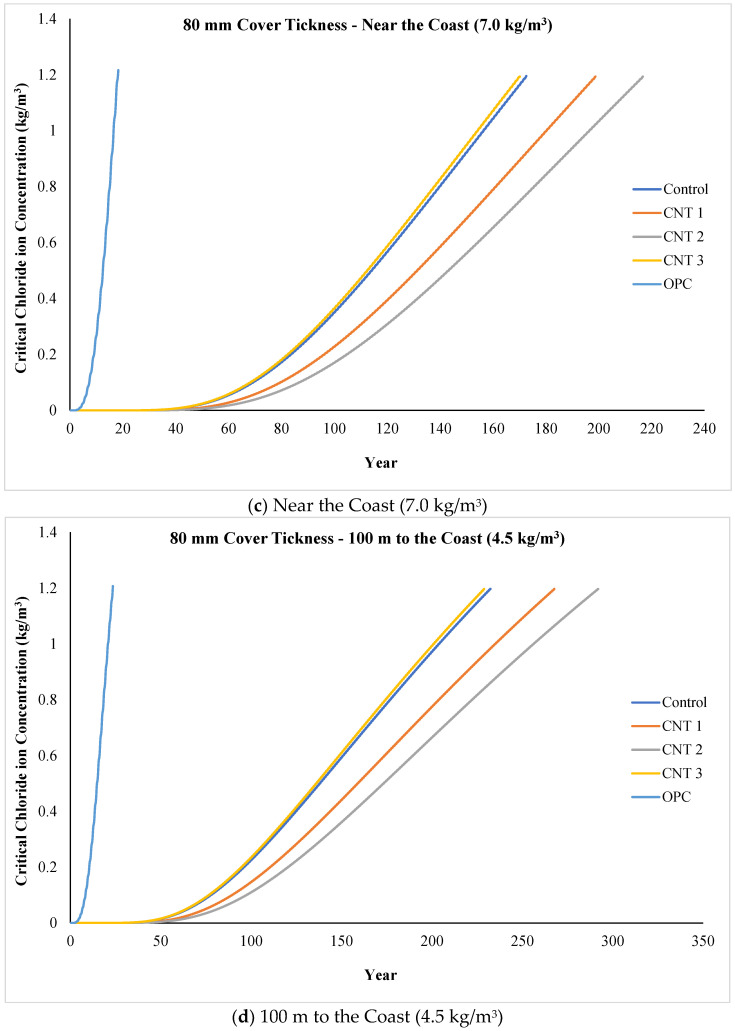

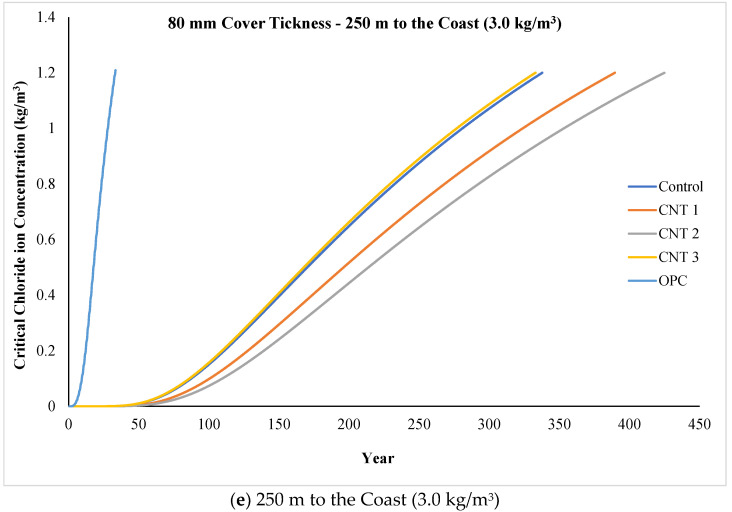
Life service prediction for all mixtures in various environmental conditions including (**a**) tidal zone, (**b**) splash zone, (**c**) near to the coast, (**d**) 100 m to the coast, and (**e**) 250 m to the coast.

The life service prediction results under diverse environmental conditions, as determined from the ponding test outcomes, are depicted in Figure 12a–e, with a consistent concrete cover thickness of 80 mm in all scenarios. Notably, in the most challenging environmental setting, the tidal zone featuring a chloride ion concentration of 17 kg/m^3^ (as shown in Figure 12a), the life service prediction model indicates ultimate resistance ages of 119, 136, 147, 118, and 19 years for the control, CNT1, CNT2, CNT3, and OPC mixtures, respectively, against chloride ions penetration. In practical terms, this implies that, in the tidal zone, all samples, excluding the OPC mix (included for comparative purposes), exhibit durability against chloride ion attack for over 100 years. Further analysis of the data underscores the superior performance of the CNT1 and CNT2 mixtures in the tidal zone. Noteworthy is the observation that, for CNT1 and CNT2 mixtures, it requires 136 and 147 years, respectively, before chloride ions can breach the concrete cover thickness of 80 mm to reach the internal reinforcement.

Derived from the life service prediction results and referencing Figure 12b, representing the second most challenging condition (splash zone with a chloride ion concentration of 7.5 kg/m^3^), the control, CNT1, CNT2, CNT3, and OPC mixtures exhibit ultimate ages of 172, 197, 216, 170, and 24 years, respectively, in their resistance against chloride ion attack. In a similar vein, noteworthy is the exceptional performance of CNT1 and CNT2, displaying longevity of around 200 years in resisting chloride ions, surpassing the other mixtures. It is essential to highlight that the OPC result is drawn from prior research and is included for comparative purposes.

Illustrated in Figure 12c are the outcomes for the near-coast condition, featuring a chloride ion concentration of 7.0 kg/m^3^ for all mixtures in this investigation. The ultimate resistance age against chloride ions penetration, as indicated by the life service prediction model, for the control, CNT1, CNT2, CNT3, and OPC mixtures in this near-coast setting were 179, 205, 223, 176, and 25 years, respectively. These findings suggest that, in terms of performance, there is minimal disparity between the splash zone and the near-coast area, given that the chloride ion concentrations in these areas were 7.5 and 7.0 kg/m^3^, respectively. Once again, CNT1 and CNT2 emerge as the top performers among all mixtures in this particular condition.

Figure 12d illustrates the life service prediction results in an environmental condition situated 100 m from the coast, with a chloride ion concentration of 4.5 kg/m^3^. According to these outcomes, the ultimate resistance age against chloride ion penetration for the control, CNT1, CNT2, CNT3, and OPC mixtures in the 100-m coastal condition were 239, 274, 298, 235, and 30 years, respectively. Furthermore, Figure 12e represents the outcomes in the most favorable environmental condition, located 250 m from the coast, with a chloride ion concentration of 3.0 kg/m^3^. In this scenario, the ultimate resistance age against chloride ion penetration for the control, CNT1, CNT2, CNT3, and OPC mixtures were 344, 396, 431, 339, and 40 years, respectively. Notably, in both scenarios, CNT1 and CNT2 exhibited superior performance compared to all other mixtures.

In summary, the life service prediction results align closely with the strength performance outcomes for all mixtures. Consistently, CNT1 and CNT2 mixtures exhibited superior performance in both compressive and tensile strength, as well as life service prediction. This can be attributed to the effective dispersion of CNT in these two mixtures, fostering the creation of additional bridges between the cement paste and ALA and MHS particles. Consequently, this contributes to the development of a more impermeable concrete matrix. As a result, these two mixtures demonstrated greater resistance against chloride ion penetration compared to the other formulations.

Ultimately, considering the required cover thickness (mm) for 200 years, detailed in Table 8, observations showed that in the tidal zone (the most challenging environmental condition), the necessary cover thickness for control, CNT1, CNT2, CNT3, and OPC mixtures were 98.71 mm, 91.90 mm, 87.99 mm, 99.49 mm, and 325.07 mm, respectively. Similarly, in the second-worst environmental condition (splash zone), the requisite cover thickness for the aforementioned mixtures was 66.72 mm, 62.11 mm, 59.47 mm, 67.25 mm, and 219.72 mm, respectively. It’s noteworthy that an enhancement in the environmental condition resulted in a reduced required cover thickness for all mixtures, aligning with the positive correlation observed with service life prediction results. Conversely, in the near-coast condition, the necessary cover thickness for control, CNT1, CNT2, CNT3, and OPC mixtures were 63.98 mm, 59.56 mm, 57.03 mm, 64.48 mm, and 210.69 mm, respectively. Furthermore, at 100 m from the coast, the values for the same mixtures were 47.53 mm, 44.25 mm, 42.37 mm, 47.90 mm, and 156.51 mm, respectively. Lastly, in the optimal environmental condition (250 m from the coast), the mandated cover thickness for the same mixtures was 32.45 mm, 30.21 mm, 28.92 mm, 32.70 mm, and 106.85 mm, respectively.

## 5. Conclusions

This study explored the mechanical characteristics, microstructure, chloride ion permeability, and service life prediction of nano-concrete utilizing ALA and MHS as a replacement for fine aggregate, along with the addition of CNT as an admixture. The impact of diverse environmental conditions on the performance of CNT concrete mixtures was systematically examined. The evaluation of CNT mixtures involved an analysis of compressive and tensile strength, XRD, SEM, accelerated chloride penetration, and service life prediction. Based on the findings presented, the following conclusions can be derived:

1. Elevating the concentration of CNT in CNT1 and CNT2 blends resulted in a marginal enhancement in compressive strength compared to the control mix. Conversely, the CNT3 mixture demonstrated a reduction in compressive strength with an increasing content of CNT as an admixture. Furthermore, the compressive strength values at 3, 7, and 28 days in the salty curing environment were significantly inferior to those recorded under air curing periods.
2. The outcomes of tensile strength testing revealed that as the quantity of CNT increased as an admixture, the tensile strength of CNT mixtures experienced a slight decline across all curing periods. This phenomenon can be ascribed to the nuanced interaction between CNTs and the cementitious matrix. While CNTs have the potential to enhance compressive strength by acting as additional bridges and reinforcing the concrete structure, the nature of their bonding with the cementitious matrix plays a critical role in load transfer. In instances where this bonding is not optimally established, a reduction in tensile strength occurs, as observed in the present study.
3. Examination through SEM indicated that an augmented concentration of CNTs as an admixture in the concrete led to the formation of nanoscale bridges within the concrete matrix. These bridges, at the nanoscale level, act as reinforcement, establishing a network within the cementitious matrix. This network plays a crucial role in significantly enhancing the distribution and transfer of loads, thereby slightly improving the overall strength performance of the concrete during both the 3-day and 28-day normal curing periods. Furthermore, an analysis of the EDS map sum spectrum outcomes revealed that the primary elements in the structure of all mixtures include O, Si, C, and Ca, albeit in varying proportions.
4. Drawing insights from the XRD analysis outcomes, it is discerned that, across all samples, the principal crystalline phases identified after both the 3-day and 28-day curing periods consist of Quartz and Alite. This occurrence can be attributed to the potential presence of certain materials in the mix in an amorphous or non-crystalline form, a condition where XRD exhibits diminished sensitivity. Furthermore, a well-homogenized mix design may result in the prevalence of dominant crystalline phases, presenting difficulties in discerning minor phases.
5. Upon scrutinizing the ponding test results, it was evident that, for all samples, the effective chloride transport coefficient remained below the standard limitation of 1.00 × 10^−12^ m^2^/s, indicating that the performance of all samples in the ponding test fell within an acceptable range. However, upon a more detailed examination, it was noted that the chloride transport coefficient experienced a decrease with an increase in the amount of CNT up to 0.05% (as observed in CNT1 and CNT2 mixes in comparison to the control mix). In contrast, for the CNT3 mixture, there was an increase relative to the control mix. This observed trend aligned with the compressive strength outcomes, where the strength of the CNT1 and CNT2 mixtures surpassed that of the CNT3 mixture.
6. The life service prediction outcomes indicate that in every environmental scenario, CNT1 and CNT2 mixtures demonstrated superior performance when compared to all other mixtures. These findings closely mirror the strength performance results, where CNT1 and CNT2 mixtures showcased excellence in both compressive strength and life service prediction. The effective dispersion of CNT in these two mixtures is credited for this superior performance, facilitating the formation of additional bridges between the cement paste and ALA and MHS particles resulting in the creation of more impermeable concrete.

## Figures and Tables

**Table 1:** Chemical composition of MHS, OPC, and SPW.

Chemical Component	SiO_2_	Fe_2_O_3_	Al_2_O_3_	TiO_2_	CaO	MgO	SO_3_	MnO	ZnO_2_	Na_2_O	P_2_O_5_	K_2_O
MHS	81.34	0.04	0.17	0.03	13.3	0.06	0.2	0.01	0.02	4.02	0.81	0.02
OPC	18.78	2.76	4.72	0.31	65.97	3.14	2.56	0.13	0	0	0.19	0
SPW	99.59	0.023	0.315	0.042	0.013	0.006	0	0	0	0.008	0	0.004

**Table 2:** Physical properties of MHS.

Physical Properties of MHS	
Shape	A hollow sphere with thin walls
Composition	Soda-lime borosilicate glass
Color	pale white powder
Pressure resistance (90% survival)	18,000 psi
Medium particle size	30 microns
Softening point	600 °C (1112 °F)
Specific Gravity	0.6
Thermal conductivity	0.05–0.20 W·m-1·K-1 @ 20 °C
Permittivity (@100 MHz)	1.2–1.9

**Table 3:** Development of Mix Design.

Lable	Water (kg)	Binder		Aggregate			CNT (kg)	SP (kg)
		OPC (kg)	SF (kg)	SPW (kg)	MHS (kg)	ALA (kg)		
Control	212.5	739	111	208	211	111	0	7.85
CNT 1	212.5	739	111	208	211	111	0.21	7.85
CNT 2	212.5	739	111	208	211	111	0.43	7.85
CNT 3	212.5	739	111	208	211	111	0.64	7.85

**Table 4:** Suggested surface chloride ion concentration in the sea [55].

Coastline	Expose Condition	Surface Chloride Concentration (kg/m^3^)
**West and** **south coast**	**Tidal Zone**	**17.0**
	**Splash Zone**	**7.5**
	Near the coast	5.0
East coast	Tidal & Splash Coast	13.0
	Near the coast	7.0

**Table 5:** Suggested surface chloride ion concentration near the sea [55].

Coastline	Expose Condition	Surface Chloride Concentration (kg/m^3^)
West and south coast	Near the coast	5.0
	100 m	2.0
	250 m	1.5
**East coast**	**Near the coast**	**7.0**
	**100 m**	**4.5**
	**250 m**	**3.0**
	500 m	2.5
	1000 m	1.5

**Table 6:** Suggested surface chloride ion concentration near the sea.

Ref.	w/b Ratio	Compressive Strength (MPa)		Tensile Strength (MPa)	
		3 Days	28 Days	3 Days	28 Days
This study	0.25	100	105	6.5	7.0
[6]	0.18	80	130	-	-
[7]	0.225	100	139	-	-
[9]	0.27	55	-	6	-
[11]	0.18	-	180	-	8
[13]	0.18	80	140	-	-
[14]	0.22	60	120	5.8	6.3

**Table 7:** Ponding test results (NT Build 443).

Sample	C_s	C_i	D_a	t
Control	0.78	0.037	8.18 × 10^−13^	90
CNT 1	0.813	0.037	7.1 × 10^−13^	90
CNT 2	0.758	0.037	6.5 × 10^−13^	90
CNT 3	0.862	0.037	8.3 × 10^−13^	90

**Table 8:** Required cover thickness (mm) over 200 years in different exposing conditions.

Expose Condition	Tidal Zone	Splash Zone	Near the Coast	100 m	250 m
Control (mm)	98.71	66.72	63.98	47.53	32.45
CNT 1 (mm)	91.90	62.11	59.56	44.25	30.21
CNT 2 (mm)	87.99	59.47	57.03	42.37	28.92
CNT 3 (mm)	99.49	67.25	64.48	47.90	32.70
OPC (mm)	325.07	219.72	210.69	156.51	106.85

## Data Availability

Data will be made available on request.

## References

[1] Saleh S., Mahmood A.H., Hamed E., Zhao X.L. (2023). The mechanical, transport and chloride binding characteristics of ultra-high-performance concrete utilising seawater, sea sand and SCMs. Constr. Build. Mater..

[2] Bogas J.A., Gomes A. (2015). Non-steady-state accelerated chloride penetration resistance of structural lightweight aggregate concrete. Cem. Concr. Compos..

[3] Cai R., Tian Z., Ye H. (2022). Durability characteristics and quantification of ultra-high strength alkali-activated concrete. Cem. Concr. Compos..

[4] Rafieizonooz M., Mirza J., Salim M.R., Hussin M.W., Khankhaje E. (2016). Investigation of coal bottom ash and fly ash in concrete as replacement for sand and cement. Constr. Build. Mater..

[5] Rafieizonooz M., Khankhaje E., Rezania S. (2022). Assessment of environmental and chemical properties of coal ashes including fly ash and bottom ash, and coal ash concrete. J. Build. Eng..

[6] Luo Z., Zhi T., Liu X., Yin K., Pan H., Feng H., Song Y., Su Y. (2023). Effects of different nanomaterials on the early performance of ultra-high performance concrete (UHPC): C–S–H seeds and nano-silica. Cem. Concr. Compos..

[7] Yu L., Bai S., Guan X. (2023). Effect of graphene oxide on microstructure and micromechanical property of ultra-high performance concrete. Cem. Concr. Compos..

[8] Song B., Liu S., Hu X., Ouyang K., Li G., Shi C. (2022). Compressive strength, water and chloride transport properties of early CO_2_-cured Portland cement-fly ash-slag ternary mortars. Cem. Concr. Compos..

[9] Afzal M.T., Khushnood R.A. (2021). Influence of carbon nano fibers (CNF) on the performance of high strength concrete exposed to elevated temperatures. Constr. Build. Mater..

[10] Oh T., Chun B., Lee S.K., Kim G.W., Banthia N., Yoo D.-Y. (2024). Effect of high-volume substituted nanosilica on the hydration and mechanical properties of Ultra-High-Performance Concrete (UHPC). Cem. Concr. Res..

[11] Zhang G., Peng G.F., Zuo X.Y., Niu X.J., Ding H. (2023). Adding hydrated lime for improving microstructure and mechanical properties of mortar for ultra-high performance concrete. Cem. Concr. Res..

[12] Wang X., Yu R., Song Q., Shui Z., Liu Z., Wu S., Hou D. (2019). Optimized design of ultra-high performance concrete (UHPC) with a high wet packing density. Cem. Concr. Res..

[13] Jiang J., Qin J., Chu H. (2023). Improving mechanical properties and microstructure of ultra-high-performance lightweight concrete via graphene oxide. J. Build. Eng..

[14] Esmaeili J., Romouzi V., Kasaei J., Andalibi K. (2023). An investigation of durability and the mechanical properties of ultra-high performance concrete (UHPC) modified with economical graphene oxide nano-sheets. J. Build. Eng..

[15] Rosa R.E.M., Corr D.J., Espinosa H.D., Shah S.P. (2023). Characterization of adhesion strength between carbon nanotubes and cementitious materials. Cem. Concr. Compos..

[16] Hussain A., Xiang Y., Yu T., Zou F. (2022). Nanocarbon black-based ultra-high-performance concrete (UHPC) with self-strain sensing capability. Constr. Build. Mater..

[17] Zhang P., Su J., Guo J., Hu S. (2023). Influence of carbon nanotube on properties of concrete: A review. Constr. Build. Mater..

[18] Narasimman K., Jassam T.M., Velayutham T.S., Yaseer M.M.M., Ruzaimah R. (2020). The synergic influence of carbon nanotube and nanosilica on the compressive strength of lightweight concrete. J. Build. Eng..

[19] Li L., Wang B., Hubler M.H. (2022). Carbon nanofibers (CNFs) dispersed in ultra-high performance concrete (UHPC): Mechanical property, workability and permeability investigation. Cem. Concr. Compos..

[20] Li C., Li J., Ren Q., Zheng Q., Jiang Z. (2023). Durability of concrete coupled with life cycle assessment: Review and perspective. Cem. Concr. Compos..

[21] Li K., Han J., Wang S., Lian H., Xiong J., Wang J., Fan Z., Xu L., Zhu H. (2023). Long-term performance of structural concretes in China southeast coastal environments exposed to atmosphere and chlorides. Cem. Concr. Res..

[22] Jung M., Lee Y.S., Hong S.G., Moon J. (2020). Carbon nanotubes (CNTs) in ultra-high performance concrete (UHPC): Dispersion, mechanical properties, and electromagnetic interference (EMI) shielding effectiveness (SE). Cem. Concr. Res..

[23] Jayakumari B.Y., Swaminathan E.N., Partheeban P. (2023). A review on characteristics studies on carbon nanotubes-based cement concrete. Constr. Build. Mater..

[24] D’Alessandro A., Ubertini F., Materazzi A.L., Laflamme S., Porfiri M. (2015). Electromechanical modelling of a new class of nanocomposite cement-based sensors for structural health monitoring. Struct. Health Monit..

[25] Aydın A.C., Nasl V.J., Kotan T. (2018). The synergic influence of nano-silica and carbon nano tube on self-compacting concrete. J. Build. Eng..

[26] Yoo D.Y., Oh T., Banthia N. (2022). Nanomaterials in ultra-high-performance concrete (UHPC)—A review. Cem. Concr. Compos..

[27] Mudasir P., Naqash J.A. (2023). Impact of carbon Nano tubes on fresh and hardned properties of conventional concrete. Mater. Today Proc..

[28] Shen P., Lu L., He Y., Wang F., Hu S. (2019). The effect of curing regimes on the mechanical properties, nano-mechanical properties and microstructure of ultra-high performance concrete. Cem. Concr. Res..

[29] Jung M., Park J.S., Hong S.G., Moon J. (2020). Micro- and meso-structural changes on electrically cured ultra-high performance fiber-reinforced concrete with dispersed carbon nanotubes. Cem. Concr. Res..

[30] Shafikhani M., Chidiac S.E. (2019). Quantification of concrete chloride diffusion coefficient—A critical review. Cem. Concr. Compos..

[31] De Weerdt K., Wilson W., Machner A., Georget F. (2023). Chloride profiles—What do they tell us and how should they be used?. Cem. Concr. Res..

[32] Sanjuán M.Á., Rivera R.A., Martín D.A., Estévez E. (2022). Chloride Diffusion in Concrete Made with Coal Fly Ash Ternary and Ground Granulated Blast-Furnace Slag Portland Cements. Materials.

[33] Dong H., Wang J. (2023). Carbon nanofibers and polyvinyl-alcohol fiber hybrid-reinforced high-performance concrete: Mechanical property, chloride penetration resistance, and material characterization. Constr. Build. Mater..

[34] Liu Q.F., Cai Y., Peng H., Meng Z., Mundra S., Castel A. (2023). A numerical study on chloride transport in alkali-activated fly ash/slag concretes. Cem. Concr. Res..

[35] Lehner P., Konečný P., Ponikiewski T. (2020). Comparison of material properties of scc concrete with steel fibres related to ingress of chlorides. Crystals.

[36] Dehghan A., Peterson K., Riehm G., Bromerchenkel L.H. (2017). Application of X-ray microfluorescence for the determination of chloride diffusion coefficients in concrete chloride penetration experiments. Constr. Build. Mater..

[37] (1995). Concrete, Hardened: Accelerated Chloride Penetration (NT BUILD 443).

[38] (2019). Standard Test Method for Determining the Penetration of Chloride Ion into Concrete by Ponding (Withdrawn 2019).

[39] (2016). Standard Test Method for Determining the Apparent Chloride Diffusion Coefficient of Cementitious Mixtures by Bulk Diffusion.

[40] (1999). Nordic Cooperation, Concrete, Mortar and Cement-Based Repair Materials: Chloride Migration Coefficient from Non-Steady-State Migration Experiments (NT BUILD 492).

[41] Mapa D.G., Zhu H., Nosouhian F., Shanahan N., Riding K.A., Zayed A. (2023). Chloride binding and diffusion of slag blended concrete mixtures. Constr. Build. Mater..

[42] Elfmarkova V., Spiesz P., Brouwers H.J.H. (2015). Determination of the chloride diffusion coefficient in blended cement mortars. Cem. Concr. Res..

[43] Runci A., Provis J., Serdar M. (2022). Microstructure as a key parameter for understanding chloride ingress in alkali-activated mortars. Cem. Concr. Compos..

[44] Lim T., Lee J.H., Mun J.H., Yang K.H., Ju S., Jeong S.M. (2020). Enhancing functionality of epoxy–TiO_2_-embedded high-strength lightweight aggregates. Polymers.

[45] Sun S., Han B., Jiang S., Yu X., Wang Y., Li H., Ou J. (2017). Nano graphite platelets-enabled piezoresistive cementitious composites for structural health monitoring. Constr. Build. Mater..

[46] Ubertini F., Materazzi A.L., D’Alessandro A., Laflamme S. (2014). Natural frequencies identification of a reinforced concrete beam using carbon nanotube cement-based sensors. Eng. Struct..

[47] García-Macías E., D’Alessandro A., Castro-Triguero R., Pérez-Mira D., Ubertini F. (2017). Micromechanics modeling of the uniaxial strain-sensing property of carbon nanotube cement-matrix composites for SHM applications. Compos. Struct..

[48] Siahkouhi M., Razaqpur G., Hoult N.A., Baghban M.H., Jing G. (2021). Utilization of carbon nanotubes (CNTs) in concrete for structural health monitoring (SHM) purposes: A review. Constr. Build. Mater..

[49] Qu Z.Y., Yu Q.L., Brouwers H.J.H. (2018). Relationship between the particle size and dosage of LDHs and concrete resistance against chloride ingress. Cem. Concr. Res..

[50] Guignone G.C., Vieira G.L., Zulcão R., Mion G., Baptista G. (2019). Analysis of the chloride diffusion coefficients by different test methods in concrete mixtures containing metakaolin and high-slag blast-furnace cement. Rev. Mater..

[51] Stanish K., Hooton D., Thomas M. (1997). Testing the Chloride Penetration Resistance of Concrete: A Literature Review.

[52] (1996). Concrete, Hardened: Chloride Content by Volhard Titration (NT BUILD 208).

[53] Ehlen M.A., Thomas M.D.A., Bentz E.C. (2009). Life-365 Service Life Prediction ModelTM Version 2.0. Concr. Int..

[54] Moffatt E.G., Thomas M.D.A., Fahim A. (2017). Performance of high-volume fly ash concrete in marine environment. Cem. Concr. Res..

[55] (2021). Concrete Structure Durability Design Standards.

[56] Sun Z., Wan S.G. (2023). Sachs, Compatibility of carbon nanotubes in concrete with air entrainer and superplasticizer. Constr. Build. Mater..

[57] Liang T., Zhou J., Wu Q. (2023). Experimental investigation on leaching behavior of ultra-high performance concrete submitted to a flow environment. Constr. Build. Mater..

[58] Xu Z., Liu Q., Long H.Y., Deng H., Chen Z., Hui D. (2023). Influence of nano-SiO_2_ and steel fiber on mechanical and microstructural properties of red mud-based geopolymer concrete. Constr. Build. Mater..

[59] Han K., Guo T., Shu X., Ran Q., Guo Y., Shi J. (2024). Insight into the role of early C3A hydration in structural build-up of cement paste. Cem. Concr. Res..

[60] Bao J., Zheng R., Zhang P., Cui Y., Xue S., Song Q., Ma Y. (2023). Thermal resistance, water absorption and microstructure of high-strength self-compacting lightweight aggregate concrete (HSSC-LWAC) after exposure to elevated temperatures. Constr. Build. Mater..

[61] Liu C., Zhang M. (2023). Microstructure-based modelling of chloride diffusivity in non-saturated cement paste accounting for capillary and gel pores. Cem. Concr. Res..

[62] Moon J., Bae S., Celik K., Yoon S., Kim K.-H., Kim K.S., Monteiro P.J.M. (2014). Characterization of natural pozzolan-based geopolymeric binders. Cem. Concr. Compos..

